# An outbreak of infection due to severe acute respiratory corona virus-2 in a neonatal unit from a low and middle income setting

**DOI:** 10.3389/fped.2022.933982

**Published:** 2022-07-28

**Authors:** Firdose Lambey Nakwa, Reenu Thomas, Alison van Kwawegen, Nandi Ntuli, Karabo Seake, Samantha Jane Kesting, Noela Holo Bertha Kamanga, Dikeledi Maureen Kgwadi, Neema Chami, Tshiamo Mogajane, Claude Ondongo-Ezhet, Thulisile Nelly Maphosa, Stephanie Jones, Vicky Lynne Baillie, Shabir Ahmed Madhi, Sithembiso Velaphi

**Affiliations:** ^1^Department of Paediatrics and Child Health, School of Clinical Medicine, Faculty of Health Sciences, University of the Witwatersrand, Johannesburg, South Africa; ^2^South African Medical Research Council, Vaccines and Infectious Diseases Analytics Research Unit, Faculty of Health Sciences, University of the Witwatersrand, Johannesburg, South Africa; ^3^African Leadership in Vaccinology Expertise, Faculty of Health Sciences, University of the Witwatersrand, Johannesburg, South Africa

**Keywords:** neonate, SARS-CoV-2, outbreak, langaroo mother care (KMC), non-pharmaceutical interventions (NPI)

## Abstract

**Introduction:**

The provision of kangaroo mother care (KMC) involving continuous skin-to-skin care (SSC) is an important intervention in neonatal care, which is recommended even when women are infected with severe acute respiratory syndrome coronavirus (SARS-CoV-2). We report on a nosocomial outbreak of SARS-CoV-2 infections in a KMC ward.

**Methods:**

Contact tracing was conducted following the diagnosis of SARS-CoV-2 in a mother lodging in the KMC ward. All mother-newborn dyads in the KMC and healthcare workers (HCW) were tested for SARS-CoV-2 within 24–72 h of diagnosing the index case. Nasopharyngeal swab samples were obtained and tested from contacts, with a nucleic acid amplification test (NAAT) assay. Next-generation sequencing was done on positive samples. The secondary attack rate (SAR) was calculated assuming that the mother who presented with symptoms was the source of infection.

**Results:**

Twelve (70.6%) of 17 mothers and 8 (42.1%) of 19 neonates who were in the KMC ward with the index case were found to be positive with SARS-CoV-2. Seven (87.5%) of the 8 neonates who tested positive had mothers who also tested positive. Seventy-five percent (9/12) of the mothers and 62.5% (5/8) of the neonates who tested positive were asymptomatic. Eight (27.6%) of 29 HCW were found to be positive and were all asymptomatic. One neonate died from *Acinetobacter baumannii* sepsis, and his post-mortem lung histopathology showed features compatible with SARS-CoV-2 pneumonia. The sequencing of 13 specimens, which included 1 mother-newborn dyad, indicated clustering to the same phylogenetic lineage with identical mutations. In assessing for factors contributing to this outbreak, it was found that spaces between beds were less than 1 m and mothers had their meals around the same table at the same time.

**Conclusion:**

We report on a nosocomial outbreak of SARS-CoV-2 in a KMC ward, affecting a high number of mothers and neonates, and to a lesser extent HCWs. Although it is difficult to point to the index case as the source of this outbreak, as asymptomatic individuals can spread infection, the inadequate adherence to non-pharmaceutical interventions was assessed to have contributed to the spread of infection. This highlights the need for awareness and adherence to mitigation strategies to avoid SARS-CoV-2 outbreaks.

## Introduction

Respiratory droplets and aerosols are regarded as part of a continuum and represent the primary mode of transmission of severe acute respiratory syndrome coronavirus 2 (SARS-CoV-2). Smaller aerosol particles (<5 microns) may travel greater distances than the larger particles (>5 microns) and have been the mode of transmission in confined close contact spaces. A number of reports have shown a higher concentration of the SARS-CoV-2 closest to the infected or the presymptomatic index cases (1). Fomites, beds, computers, and bed rails have been identified as sources of transmission. Hospital areas frequented by medical staff had a higher yield of the virus than patient-accessible areas (1, 2). Hospital- or nosocomial-acquired severe acute respiratory syndrome coronavirus 2 (SARS-CoV-2) infection has been reported early in the course of the pandemic, with one South African hospital, where 6 clusters with 119 phylogenetically linked infections were identified, and 15 deaths were reported (3).

In a meta-analysis by Haley et al., the hospital-acquired secondary attack rate (SAR) varied from 0 to 17.1% with a pooled estimate of 3.6% (95% CI 1.0–6.9) and no difference between the patient population and the heathcare workers (HCWs). Household contacts had higher SAR than hospital-acquired infections. The SAR was highest in pre-symptomatic and symptomatic cases than in asymptomatic cases (4).

Neonates may be infected by SARS-CoV-2 rarely *via* vertical transmission *in utero* (5–7) and more commonly through airborne, droplet, and contact spread from their mothers and HCWs (8). In a recent systematic review, a pooled estimate of 3.2% of neonates born to women infected by SARS-CoV-2 at the time of delivery were infected during the perinatal period (9). A higher positivity rate in neonates has been reported in low- and middle-income countries (LMICs) at 5.1% compared to 3.8% in HIC (10). Thirteen percent of neonates tested positive in the INTERCOVID multinational study with data from 18 countries (11). Studies detailing pregnancy and neonatal outcomes are small case series and case reports. The small case series representing pooled data from LMICs such as Bangladesh, India, Nigeria, and Iran had reported a low positivity rate in neonates born to SARS-CoV-2 positive mothers (12–16).

Neonates may also be infected during community outbreaks or family cluster outbreaks (17–19). Nosocomial SARS-CoV-2 infection has been reported in adults (20), but there are few reports of nosocomial infections in neonates from LMIC (21–27). In national population studies in the United Kingdom and Spain, nosocomial-acquired cases were identified in 8/66 (12%) and 14/40 (35%) of positive neonates. The Spanish study was a prospective observational study in 79 hospitals with 26 community- and 14 nosocomial-acquired cases in neonates over an 8-week period. The median age at diagnosis in the nosocomial acquired cases was 14.5 (7.2–43 days) compared to 17 (11.5–26.5) days in the community-acquired cohort. The mothers and HCWs were considered to be possible sources of infection in 12 neonates (8). The United Kingdom study was a population-based study in SARS-CoV-2-positive neonates in the first 28 days over an 8-week period in 155 hospitals. Sixty-six neonates were positive, giving an incidence of 5.6%, with 17 neonates born to SARS-CoV-2-positive mothers and 8 neonates who had SARS-CoV-2 from the nosocomial transmission. Of these, 6 were preterm and acquired the infection >7 days after birth. The median age at diagnosis was 9.5 (7.5–11.0) days. Half of the neonates who were SARS-CoV-2 positive had family members and close contacts who were also positive (28). Most of the neonates in both these studies were preterm with favorable outcomes and had neonatal comorbidities (8, 28).

In this report, we present findings of an outbreak of maternal and neonatal SARS-CoV-2 infections that occurred in a kangaroo mother care (KMC) ward in a public, tertiary hospital in a low- and middle-income setting.

## Materials and methods

An investigation for SARS-CoV-2 infection of all mother-newborn dyads lodging in a 24-h KMC at Chris Hani Baragwanath Academic Hospital (CHBAH), and healthcare workers, was conducted after one of the women tested positive for SARS-CoV-2 infection following investigation for upper respiratory symptoms. CHBAH is a tertiary public hospital in Johannesburg, South Africa. It has a total of 3,200 beds, including a 185-bed neonatal unit consisting of 18 intensive care, 48 high care, 100 standard care, and 19 KMC beds. The daily bed occupancy is generally more than 80%.

The KMC ward is a facility for otherwise well preterm-born neonates who are awaiting weight gain, and their mothers are encouraged to lodge in the ward to offer skin-to-skin care (SSC) to their babies throughout the day. Neonates admitted to the KMC include those weighing between 1,000 and 1,700 g, not requiring supplemental oxygen or intravenous fluids, and whose mothers are available to room-in with the baby. Mothers provide SSC continuously on their beds except when bathing or having meals, during which the babies are placed in the bassinettes next to the mothers’ bed. The ward accommodates up to 19 mother-neonate dyads.

The KMC ward is adjacent to another ward which is a standard care nursery (SCN) accommodating up to 20 neonatal admissions. Neonates admitted to SCN and KMC wards are assessed and managed by the same nursing and medical staff. Because of limited lodging facilities, women of neonates requiring hospital admission (sick and small neonates) not in the KMC ward were discharged home and could visit daily.

At the onset of the COVID-19 outbreak in South Africa, mothers were screened daily for symptoms suggestive of COVID-19 while staying in the KMC ward. On 8 June 2020, one of the mothers manifested a fever and cough and was diagnosed with COVID-19 following testing positive for SARS-CoV-2 from nasopharyngeal swabs, which was tested using a nucleic acid amplification assay test (NAAT). This mother was considered the index case for SARS-CoV-2 infection in this ward. She had been lodging in the KMC ward since 29 May 2020. She was counseled on self-monitoring and sent home to self-isolate.

Nasopharyngeal swabs were performed on all the other mothers and their babies who were residing in the KMC and investigated for SARS-CoV-2 using the NAAT assay, within 24 and 72 h after identifying the index case, respectively. The secondary attack rate (SAR) was calculated based on the probability of onward infection from an index case among a defined group of close contacts (4). Testing was also conducted on all the neonates admitted to the standard care nursery adjacent to the KMC. As part of the investigation, the specimens from mothers and babies that tested positive for SARS-CoV-2 on NAAT were sequenced by Next Generation Sequencing (NGS) at the Wits Vaccines and Infectious Diseases Analytics (Wits-VIDA) Research Unit. The cause of death attribution was undertaken by a multidisciplinary team including pediatricians, microbiologists, and pathologists, who evaluated the cause based on ante-mortem and post-mortem tests, including histology.

### Testing for severe acute respiratory corona virus-2

Total nucleic acids were extracted from the respiratory swabs using the Bioer automated extraction system together with the MagaBio Plus virus DNA/RNA purification kit II as per manufacturers’ instructions (Hangzhou Bioer Technology Co., Ltd, China). NAAT was performed using the U.S. Centers for Diseases Control and Prevention’s emergency use authorization assays to detect SARS-CoV-2 (29). Samples were determined to be positive for SARS-CoV-2 when both of the nucleocapsid targets (N1 and N2) were positive with a cycle threshold (Ct) < 40. The samples were classified as inconclusive if only a single target was detected.

### Genomic viral sequencing

Mothers and neonates who were tested SARS-CoV-2 PCR-positive underwent viral sequencing using Superscript IV with random hexamers (Life Technologies, Carlsbad, CA, United States) to generate cDNA. The Swift Biosciences’ Normalase Amplicon and the SARS-CoV-2 amplicon panel (30) were used to amplify and index paired-end libraries of the genomic DNA. The resulting libraries were sequenced using a 300 cycle v2 MiSeq Reagent Kit on an Illumina MiSeq instrument (Illumina, San Diego, CA, United States).

### Genome assembly and phylogenetic analysis

The Genome Detective 1.126 and the Coronavirus Typing Tool^1^ were used to generate paired-end fastq reads (31). The bcftools 1.7-2 mpileup method was then used to filter out the low-quality mutations, and all the sequences were deposited in GISAID.^2^ A custom pipeline based on a local version of NextStrain was used for the phylogenetic analysis using a South African dataset with genomes collected during the same time as the study period. Genomes with low sequence genome coverage (<90%) were filtered out of the pipeline which contains several python scripts that manage the analysis workflow. It performs alignment of genotypes in MAFFT, phylogenetic tree inference in IQ-Tree20, ancestral state construction, and annotation.^3^

### Data analysis

The medical records of all the mothers and neonates who were tested and had NAAT results available were reviewed for clinical characteristics and outcomes at hospital discharge. The proportion of mothers and neonates with positive SARS-CoV-2 results, including the concordance of SARS-CoV-2 results between neonates and their mothers, and between twins were assessed.

The clinical and laboratory data were collected prospectively and information on outcomes was extracted from the medical records. Descriptive stats were represented as numbers and proportions.

### Ethics approval

Permission to report on this outbreak was acquired from the hospital management, and ethics approval was obtained from the University of the Witwatersrand Human Ethics Research Committee (Protocol Number – M200505).

## Results

**FIGURE 1:**
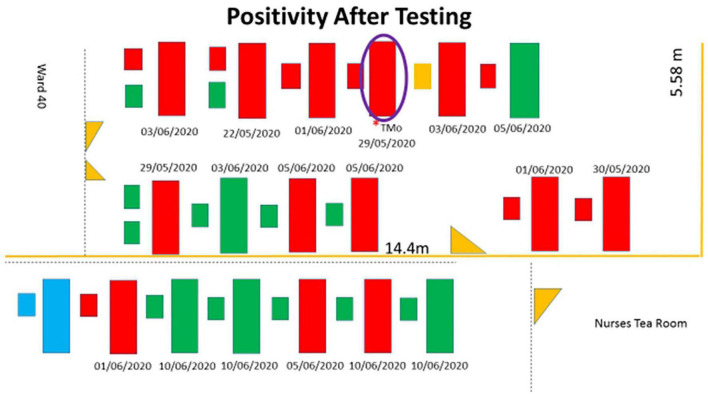
Schematic representation with the mothers and neonates who tested positive shaded in red. The beds shaded in green are mothers and neonates who tested negative, and the beds shaded in blue are not occupied. The large rectangle represents the maternal beds, the smaller rectangles adjacent to the large rectangles represent neonatal beds, and two smaller rectangles represent a set of twins. The purple circle represents the index case. The small orange rectangle represents the neonate who was not tested. Yellow triangles are doors. The dates below the beds are the dates of admission to the KMC ward.

There were 17 mothers and 20 babies (including 3 sets of twins) admitted in the KMC ward between 8 June 2020, the day the index case was investigated, and 10 June 2020 when her SARS-CoV-2 result was received. Thirteen (76%) mothers had been admitted to the ward since 29 May 2020 and four (24%) were admitted between 8 and 10 June 2020. The bed location of mother-newborn dyads who were tested following confirmation of COVID-19 in the index case is presented in Figure 1. All infants were born preterm (<36 weeks), with median birth weights of 992.5 (750–1,600) g, and the median weights at the time of the test were between 1562.5 (1470–1640) g. The median postnatal age at the time of testing was 50.5 (23–72) days. Respiratory distress syndrome was the admitting diagnosis to the neonatal unit at birth for all the neonates who were positive for SARS-CoV-2. One neonate who had underlying chronic lung disease (CLD) was weaned to room air before admission to the KMC ward. This neonate decompensated and required ventilation and subsequently demised after being infected by SARS CoV-2.

Twelve (70.6%) of 17 mothers and 8 (40%) of 20 neonates (including neonates of the index case) tested positive for SARS-CoV-2 infection (Table 1). One neonate was discharged and taken home by the mother after she was tested (she tested positive), but before her infant could be tested. Seven (87.5%) of the eight positive neonates had mothers who tested positive. Of the 12 neonates, including a set of twins, who tested negative for SARS-CoV-2, 6 (50%) of these mothers were infected with SARS-CoV-2. Overall, 20 of the 36 neonates tested after the index case was identified, were positive. If one assumes that the source of infection was the index case and considering the mother and infant as independent individuals, the overall secondary attack rate was 55.5% (20/36), and when mother-infant dyads are considered as one individual, the transmission rate was 76.5% (13/17).

**TABLE 1:** Results of COVID test and outcome of the mothers and neonates in the kangaroo mother care (KMC) ward.

COVID-19 result	Baby result	Outcome
Positive	Positive	Discharged
Positive	Positive	Discharged
Negative	Positive	Demised
Negative	Negative	Discharged
Positive	T1 Negative/T2 Positive	Discharged/discharged
Positive	T1 Positive/T2 Negative	Discharged/discharged
Positive	Negative	Discharged
Positive	Negative	Discharged
Positive	Not tested	Discharged
Positive	Positive	Discharged
Positive	Positive	Discharged
Positive	Positive	Discharged
Positive	Negative	Discharged
Positive	T1 Negative/T1 Negative	Discharged/Discharged
Negative	Negative	Discharged
Positive	Negative	Discharged
Negative	Negative	Discharged
Negative	Negative	Discharged

All the three mothers who had twins were positive for SARS-CoV-2, but only one of two sets tested positive, and both neonates of the third set of twins were negative (Table 1).

### Outcomes of severe acute respiratory corona virus-2-positive mothers and infants

Among the 12 women who tested positive and confirmed to be infected by SARS-CoV-2, nine (75%) were asymptomatic, and three had mild upper respiratory tract symptoms. All the women infected with SARS-CoV-2 were discharged upon diagnosis for self-isolation at home, and one was sent to a government isolation facility.

Three (37.5%) of the neonates who had a reactive NAAT test were symptomatic. There was a single death in one of the neonates with COVID-19 who developed nosocomial pneumonia and warranted mechanical ventilation and cultured multi-drug resistant *Acinetobacter baumannii* in blood. Post-mortem histopathological findings (13 days after the SARS-CoV-2 positive result), obtained by targeted minimal invasive tissue sampling, revealed hyaline membrane, type II pneumocyte proliferation, interstitial inflammation, markedly reactive bronchial cells, and intravascular thrombi, which could be consistent with COVID-19. The immediate cause of death was assigned to be attributed to *A. baumannii* nosocomial sepsis with the antecedent cause related to nosocomial SARS-CoV-2 and acute respiratory distress syndrome due to SARS-CoV-2 (32).

There were 13 babies in the adjacent SCN ward who were also tested, all of whom had a non-reactive NAAT for SARS-CoV-2. One of these neonates was tested positive on a subsequent visit to the pediatric casualty ward 10 days after having been discharged on 15 June 2020, and this neonate was declared to be deceased upon arrival at the hospital. The verbal autopsy revealed that the neonate had difficulty with breathing on the evening of 24 June 2020 and was taken to the family physician who sent the neonate home on unspecified treatment. The baby was noted to be unresponsive in the early hours of the morning of 25 June 2020 and brought to the hospital.

**FIGURE 2:**
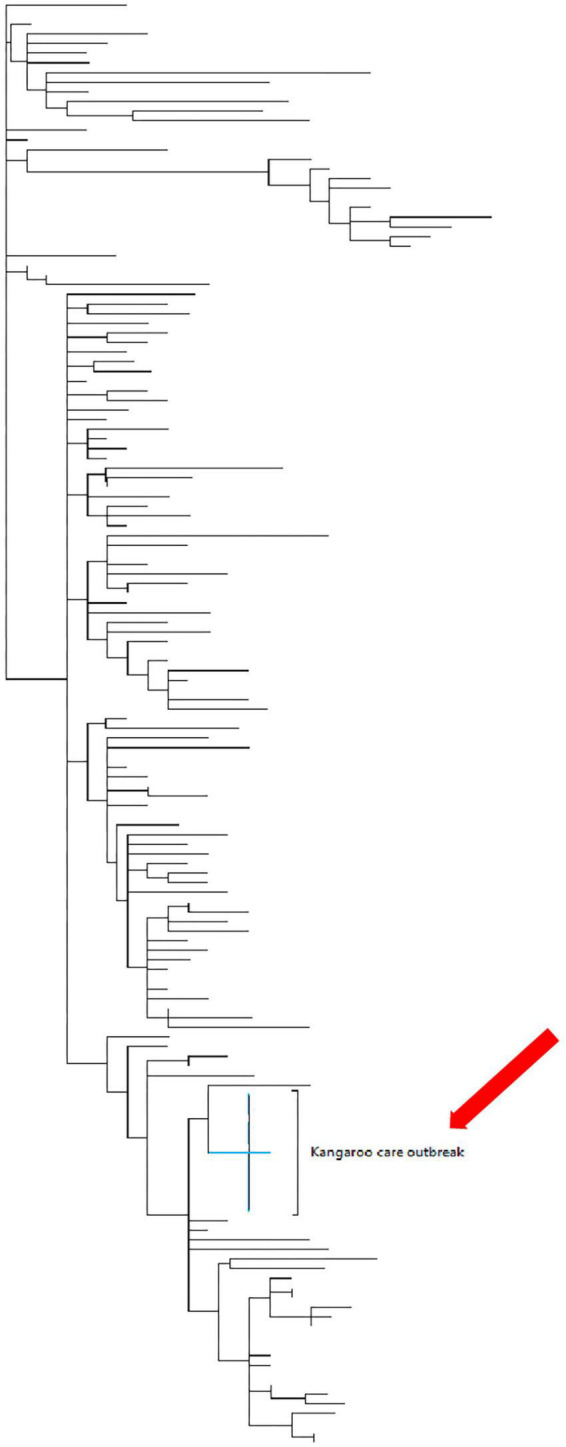
The maximum clade credibility phylogeny of the mother and babies who tested positive during the outbreak in the Kangaroo care ward. Phylogenetic tree of 160 genomes collected in SA during the time that the outbreak had occurred. The maximum clade credibility phylogeny of the mothers and neonates who were tested during the outbreak in the KMC ward (red arrow). This indicates that the genomic data obtained from the 10 mothers and 3 neonates were clustered closely together.

The next-generation sequencing was successfully performed in samples from 10 (77%) of 13 women and 3 (38%) of 8 neonates, with the remaining samples not sequenced due to low viral loads (cycle threshold >32). The sequenced samples included one mother-newborn dyad. All of the genomes were of the C.35 PANGO lineage classification; furthermore, all of the genomes clustered very closely together during the phylogenetic analysis with identical mutations at D614G and P314L positions (Figure 2). A single genome from one neonate clustered with the other outbreak genomes, albeit having several additional mutations at A831V.

Twenty-nine healthcare workers (3 doctors, 25 nurses, and 1 ward clerk) who worked in the KMC ward were also tested for SARS-CoV-2 as part of the outbreak investigation; 27.6% (8/29; 1 doctor, 6 nurses, and 1 ward clerk) had a reactive SARS-CoV-2 NAAT.

On reviewing the activities in the ward, it was identified that the mothers shared the same table for meals without observing social distancing. The mothers had been wearing cloth face masks during their stay in the ward. During the assessment of spacing between beds, the distance between the edges of the beds for mothers was recorded as 75 cm and between baby and mother was recorded as 25 cm.

## Discussion

We report on a nosocomial outbreak of SARS-CoV-2 in a KMC ward, affecting mothers and their infants. Forty percent of neonates and 70% of mothers in the KMC ward were positive during this outbreak and 27.6% of HCWs were found to be positive. Other than the index cases, most mothers and infants who tested positive were asymptomatic. Vertical transmission was considered to be unlikely as the babies in the KMC ward were chronologically older, thus, more likely to be a horizontal transmission.

A number of studies have reported on the mother-to-infant transmission of SARS-CoV-2 but most of them were around the time of birth or soon after birth, and they reported low transmission rates of less than 3.2% (9). A more recent meta-analysis reported a higher transmission rate of 12% in a study where 54 neonates were positive for SARS-CoV-2. Most of these studies reported that infants were placed 6 ft or 1.5 m from their mothers and offered intermittent SSC, but they did not report on the duration of SSC (7, 33–36). A national study from India had 21 (1.43%) neonates who tested positive beyond 72 h and reported these as horizontal transmission and had a higher positivity rate in neonates rooming in with their mothers (22). Being in a ward where there is a documented SARS-CoV-2 outbreak and documented contact with a suspected SARS-CoV-2 patient have been reported as risk factors for nosocomial SARS-CoV-2 infections (37). The rates of infection were also noted to be higher if the duration of exposure was for longer than 5 days (4). In this study, where continuous SSC was a norm, there was a high mother-to-child postnatal transmission rate or concordance rate. While the inadequate implementation of NPI most likely contributed to the spread of infection and hence the higher transmission rate, it is possible that a continuous provision of SSC by a SARS-CoV-2-positive mother increased the risk of transmission compared to intermittent SSC.

Many studies have reported low or no mortality in neonates infected by SARS-CoV-2. In this study, we report that one (12.5%) of the neonates who were positive subsequent to this outbreak died with features suggestive of SARS-CoV-2, though the immediate cause was assessed to be healthcare-associated infection due to *A. baumannii*. In reviewing this death, the histopathological changes observed were not in keeping with *A. baumannii* sepsis. The second neonate who died was in the SCN during the time of this outbreak. Though he was negative at discharge, it is possible that he acquired the infection during this outbreak as he presented within 14 days of the outbreak, but the acquisition of infection from the home or community cannot be excluded.

All the specimens sent for next-generation sequencing had the same lineage classification, and the genome of each specimen was clustered closely together on the phylogenetic tree. Thus, there was a common source for the outbreak in the KMC ward. These were representative of the common SARS-CoV-2 PANGO lineages circulating during the pandemic (38). Healthcare worker specimens were analyzed by a different laboratory and hence were not sent for NGS. Therefore, we could not conclusively state that a healthcare worker from the community was the primary source.

The response to the outbreak was to increase the space between beds from 75 to 150 cm. Mothers were offered surgical masks to wear at all times and were divided into two groups when going for meals to allow a space of 150 cm between mothers during meals. Mothers who came from home were screened for symptoms and tested for SARS-CoV-2, and only those who tested negative were admitted in the 24-h KMC ward. The positive mothers were only accepted into the KMC ward to perform 24-h KMC after they had completed 10 days of isolation at home or in a government isolation facility. There have been no other cases of COVID-19 from the KMC ward since the implementation of the above measures.

Limitations of the study are that we did not swab the environment, the beds, bed rails, bedside lockers, or any of the ablution facilities or the staff common areas. Environmental swabs and airborne samples were not taken to investigate for SARS-CoV-2. The HCW nasopharyngeal swabs were not sent for NGS testing as these were tested by a different laboratory and we had no access to these samples. Strengths are that it reports on an outbreak in a KMC ward and the importance of NPI measures and screening.

Our recommendations are that all mothers coming for labor and delivery should be screened and/or tested for SARS-CoV-2. Mothers who tested positive should be isolated, and those whose babies are well, to room-in with their babies and provide intermittent SSC during breastfeeding, keeping the 1-m distance between baby and mother in between feeds, mothers should wear surgical masks, especially during breastfeeding and practice hand hygiene. The mothers who test positive and are to remain in hospital to provide continuous SSC in the KMC ward with other negative mothers, these positive mothers to only be admitted to the KMC ward after 10 days of isolation. For mothers whose infants might have been too sick or too small for them to lodge while waiting for their infants and therefore were sent home, they must be screened for symptoms and tested for SARS-CoV-2 before readmission to the lodger facility or KMC ward. Mothers who are symptomatic and/or test positive should not be admitted to KMC; they should be referred to their family doctor or physician depending on the severity of symptoms and to be in isolation or quarantine. They should be readmitted once they have been discharged by their physicians or are de-isolated. Mothers should be encouraged to express breastmilk and ask the family members to bring the breastmilk to the hospital for the baby.

## Conclusion

This study reports on a SARS-CoV-2 outbreak in a KMC ward with mothers, neonates, and HCWs infected. The majority of the infected neonates had a good outcome in terms of respiratory morbidity. There was a low mortality rate. The outbreak highlights the importance of adhering to NPI in mitigating the spread of SARS-CoV-2 in a hospital setting with such close and prolonged contact. As we aim for zero separation of mother–newborn dyads and promote rooming-in and breastfeeding, even in mothers who are infected with SARS-CoV-2, we must impress on strategies to curtail the spread of these infections.

## Data availability statement

The raw data supporting the conclusions of this article will be made available by the authors, without undue reservation. The data presented in the study are deposited in the GISAID EpiCoV repository and the accession numbers are: EPI_ISL_12690551, EPI_ISL_12690550, EPI_ISL_12690542, EP I_ISL_12690553, EPI_ISL_12690541, EPI_ISL_12690552, EPI _ISL_12690544, EPI_ISL_12690555, EPI_ISL_12690543, EPI_I SL_12690554, EPI_ISL_12690546, EPI_ISL_12690557, EPI_IS L_12690545, EPI_ISL_12690556, EPI_ISL_12690548, EPI_ISL _12690547, and EPI_ISL_12690549.

## Ethics statement

The studies involving human participants were reviewed and approved by the University of the Witwatersrand Human Research Ethics Committee. Written informed consent from the participants’ legal guardian/next of kin was not required to participate in this study in accordance with the national legislation and the institutional requirements.

## Author contributions

FN and SV conceptualized and analyzed the data of the study and wrote the first draft. FN, RT, AK, NN, SK, KS, NK, DK, TNM, CO-E, TM, and NC collected and analyzed data. VB, SJ, and SM analyzed and interpreted the NGS data. All authors analyzed the drafts and are accountable for the content.

## Footnotes

1
https://www.genomedetective.com

2
https://www.gisaid.org/

3
https://github.com/nextstrain/ncov
